# The Non-Canonical Role of Aurora-A in DNA Replication

**DOI:** 10.3389/fonc.2015.00187

**Published:** 2015-08-25

**Authors:** Takaaki Tsunematsu, Rieko Arakaki, Akiko Yamada, Naozumi Ishimaru, Yasusei Kudo

**Affiliations:** ^1^Department of Oral Molecular Pathology, Institute of Biomedical Sciences, Tokushima University Graduate School, Tokushima, Japan

**Keywords:** Aurora-A, geminin, DNA replication, pre-RC, ubiquitin, proteasome, degradation

## Abstract

Aurora-A is a well-known mitotic kinase that regulates mitotic entry, spindle formation, and chromosome maturation as a canonical role. During mitosis, Aurora-A protein is stabilized by its phosphorylation at Ser51 via blocking anaphase-promoting complex/cyclosome-mediated proteolysis. Importantly, overexpression and/or hyperactivation of Aurora-A is involved in tumorigenesis via aneuploidy and genomic instability. Recently, the novel function of Aurora-A for DNA replication has been revealed. In mammalian cells, DNA replication is strictly regulated for preventing over-replication. Pre-replication complex (pre-RC) formation is required for DNA replication as an initiation step occurring at the origin of replication. The timing of pre-RC formation depends on the protein level of geminin, which is controlled by the ubiquitin–proteasome pathway. Aurora-A phosphorylates geminin to prevent its ubiquitin-mediated proteolysis at the mitotic phase to ensure proper pre-RC formation and ensuing DNA replication. In this review, we introduce the novel non-canonical role of Aurora-A in DNA replication.

## Introduction

Cyclin-dependent kinases (CDKs) acquire catalytic activity by forming complexes with the cyclins and promote cell cycle progression via phosphorylation of crucial target proteins (1). In mitosis, other kinases such as Aurora-A, Aurora-B, and Aurora-C tightly regulate drastic and rapid morphological changes (2). Aurora-A, the serine/threonine kinase, is essential for several events during mitosis including entry of mitosis, duplication of centrosome, spindle formation, segregation of chromosome, and cytokinesis (3). Aurora-A protein expression peaks during mitosis and decreases at G_1_ phase in mammalian cells (4). Expression of Aurora-A protein is reduced in late mitosis as a consequence of ubiquitin-mediated proteolysis by anaphase-promoting complex/cyclosome (APC/C) and its co-activator Cdh1 (5–7). It is well known that protein level of various cell cycle regulators is regulated by the ubiquitin–proteasome system (UPS) for proper regulation of cell cycle (1, 8). Aurora-A protein is ubiquitylated via recognition of destruction box (D-box) in the C-terminal by Cdh1 (5) and an additional A-box/DAD motif (9, 10). Furthermore, Ser53 (equivalent to Ser51 in human Aurora-A) of the A-box is phosphorylated during mitosis and this phosphorylation is important for the protein stabilization of *Xenopus* and human Aurora-A (4, 11, 12).

DNA replication is strictly restricted to occur only once per cell cycle in eukaryotes. To prevent over-replication, replication origins are restricted to activate only once per cell cycle by a mechanism called “licensing.” The assembly of the pre-replication complex (pre-RC) mediates licensing at the origins of replication (13, 14). The assembly of the pre-RC at replication origins can only occur from late mitosis to early G_1_ with low CDK activity and high activity of APC/C (13, 14). Once pre-RC complexes are assembled, origins are licensed for replication in the ensuing S phase. Geminin is known as a repressor of re-replication and directly binds to chromatin licensing and DNA replication factor 1 (Cdt1) to prevent pre-RC formation (15). Recently, we found that Aurora-A phosphorylates geminin to prevent its ubiquitin-mediated proteolysis at the mitotic phase to ensure proper pre-RC formation and ensuing DNA replication. In this review, we introduce the novel non-canonical role of Aurora-A in DNA replication, notably its initiation process called “licensing.”

## Ubiquitin–Proteasome Pathway

The UPS marks proteins for destruction by attaching a polyubiquitin chain and subsequently degrading these proteins via the activity of a multicatalytic enzyme, 26S proteasome (8). Ubiquitin in its monomeric form is a small protein that contains only 76 amino acids. Attachment of a polyubiquitin chain to a substrate requires the concerted action of three enzymes, E1 (ubiquitin-activating enzyme), E2 (ubiquitin-conjugating enzyme), and E3 (ubiquitin ligase) (8). E1 forms a high-energy thioester bond with ubiquitin in an ATP-dependent reaction, and then the ubiquitin molecule is transferred from E1 to E2. E3 is classified into two distinct classes based on the homology domain: HECT and RING domains. The HECT-type E3s form covalent linkages with ubiquitin from E2 by using a conserved cysteine and subsequently transfer ubiquitin to substrates. On the contrary, the RING-type E3s function as adaptors to facilitate the positioning and transfer of ubiquitin from E2 directly onto the substrate (16). A number of E3s have been found to physically bind to the substrate. Both E2 and E3 proteins exist as large families and the substrate specificity is thought to be defined by different combinations of E2s with different E3 proteins. The human genome encodes only two E1s and less than 40 E2s. Moreover, more than 600 different E3 ligases have been identified in the human genome, allowing for tremendous diversity in substrates (17).

## Cell Cycle Control by APC/C Ubiquitin Ligase

**Figure 1:**
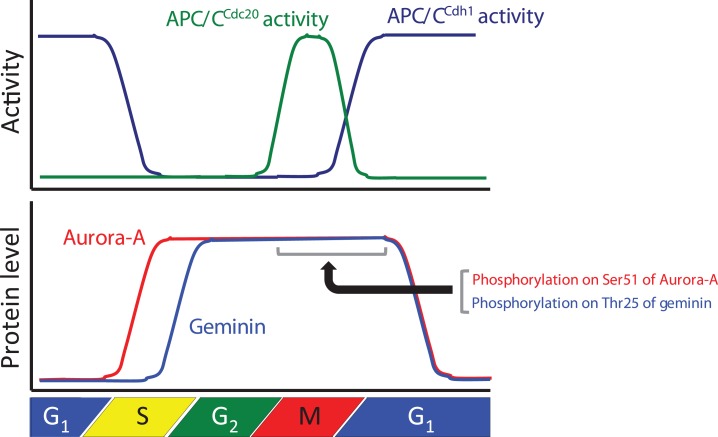
**Relationships between the protein levels of Aurora-A and geminin and anaphase-promoting complex/cyclosome (APC/C) activity during cell cycle progression**. The graph shows APC/C^Cdc20^ and APC/C^Cdh1^ activities and the protein levels of Aurora-A and geminin during cell cycle progression.

The specific, rapid, and timely proteolysis of cell cycle regulators by the UPS represents an important mechanism that ensures proper progression via the cell division cycle in a unidirectional and irreversible manner. The proteolysis of many core components of the cell cycle machinery is controlled by two major classes of ubiquitin ligases, the SCF complex and the APC/C complex, which are RING-type E3s. SCF complexes represent an evolutionarily conserved class of E3 enzymes containing four subunits: *S*kp1, *C*ul1, one of many *F*-box proteins, and Roc1/Rbx1 (18). APC/C is composed of at least a dozen different subunits, namely APC1, APC2, Cdc27/APC3, APC4 APC5, Cdc16/APC6, APC7, Cdc23/APC8, Doc1/APC10, APC11, CDC26, and APC13, but it can only ubiquitylate substrates with the help of a co-activator protein (19). In mammalian cells, APC/C activity is regulated by its binding with the co-activator proteins Cdc20 and Cdh1 during specific periods of the cell cycle (19) (Figure 1). All of these proteins are characterized by the presence of sequence elements, known as the C-box and the IR-tail, which mediate their binding to APC/C (20–22). Cdc20 and Cdh1 contain a C-terminal WD40 domain that is predicted to fold into a propeller-like structure and that is believed to recognize APC/C substrates by interacting with specific recognition elements in these substrates called D-box (RxxL) and KEN-box (KEN) (23–25). In addition to D-box and KEN-box, other motifs, including A-box (RxLxPSN), CRY-box (CRYxPS), GxEN-box (GxEN), Spo13 D-box (LxExxN), and O-box (unknown sequence), are also recognized by APC/C (10, 11, 26–29). The APC/C^Cdc20^ complex is necessary for progression through mitosis and it facilitates the exit from mitosis by inactivating CDK1, and the APC/C^Cdh1^ complex helps to maintain low CDK activity and the G_0_/G_1_ state (19, 30) (Figure 1). The APC/C^Cdh1^ and APC/C^Cdc20^ complexes target distinctive specific substrates. Although several recent studies have indicated that both co-activators and APC/C have important roles in substrate recognition, the mechanism by which APC/C recognizes its substrates is unclear. As inappropriate activation of APC/C could cause fatal errors in cell cycle progression, protein degradation via APC/C activation is tightly controlled. APC/C activation is also regulated by APC/C inhibiting proteins, such as mitotic arrest-deficient 2 (Mad2), budding uninhibited by benzimidazole-related 1 (BubR1), budding uninhibited by benzimidazole 1 (Bub1), and early mitotic inhibitor 1 and 2 (Emi1 and Emi2) (19). However, it is also unclear how the timing of degradation of numerous APC/C substrates is regulated. Indeed, substrates are not degraded at the same time by APC/C in spite of activation of APC/C during mitosis. It is unclear why the timing of the ubiquitylation of substrates is different. It was recently demonstrated that (i) phosphorylation and acetylation interfere with ubiquitylation of substrates by APC/C (4, 31–33), (ii) intrinsic regulation of APC/C by substrate ordering is attributable to kinetic differences in the ubiquitylation process (34), and (iii) ubiquitylation of the substrate is inhibited by the binding protein of APC/C (35). Thus, the timing of the ubiquitylation by APC/C may be regulated by protein modification, the processing of ubiquitylation, and binding by an inhibitor.

## Aurora-A Kinase

Aurora-A is one of the Aurora kinases (Aurora-A, Aurora-B, and Aurora-C), which are highly conserved serine/threonine kinases (36). Aurora-A plays an important role in chromosomal alignment and segregation during mitosis and meiosis (36). Indeed, Aurora-A phosphorylates a large number of substrates, including p53, polo-like kinase-1 (PLK1), CDC25B, BRCA1, centrin, LATS2, GEF-H1, TACC3, NDEL1, HDAC6, Ski, HURP, PP1, TPX2, Eg5, histone H3, CENP-A, CENP-E, CEP192, CEP192, CPEB, LIMK1, LIMK2, SRC, RalA, AKT, and PC2 (37). Aurora-A-mediated phosphorylation of substrates contributes to the activation of kinase activity, protein degradation, protein stabilization, targeting of the centrosome, maturation and separation of centrosome, translocation, and negative regulation of protein function (37). For example, phosphorylation of p53 is involved in its protein degradation (38). Aurora-A activates Plk-1 in G_2_ phase via the direct phosphorylation of Thr210 (39). Phosphorylation of LATS2, NDEL1, and TACC3 promotes centrosome maturation (40–42). Aurora-A shares high homology between species and it is evolutionarily ancient, with Aurora-A sharing 82% sequence identity between the human and rodent genes. Aurora-A contains a key threonine, the T-loop residue Thr288, within its kinase domain, and Thr 288 is phosphorylated to allow for kinase activity via autophosphorylation (9, 43, 44). The expression level of Aurora-A mRNA and protein is controlled in a cell cycle-dependent manner. Expression of Aurora-A mRNA peaks at G_2_/M, with protein expression peaking slightly later (45, 46). The promoter of Aurora-A contains specific sequences required for transcription in G_2_ phase (46–48). Expression of Aurora-A protein peaks during mitosis and decreases in G_1_ phase as a consequence of ubiquitylation by APC/C^Cdh1^ (4–7) (Figure 1).

**Figure 2:**
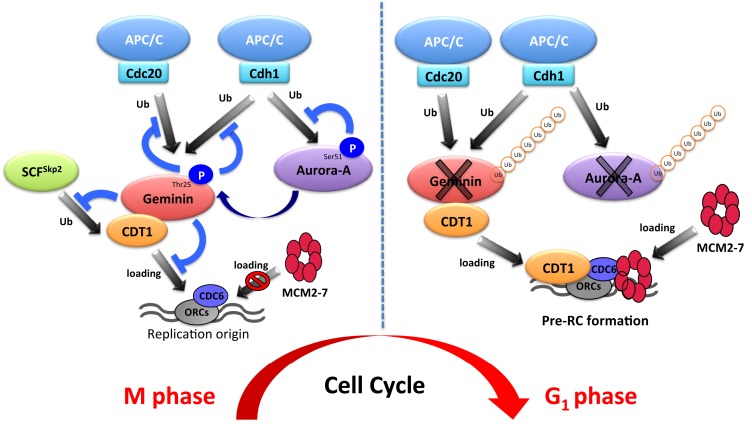
**Schematic model of DNA replication via the aurora-A–geminin–CDT1 axis**.

The APC/C^Cdh1^ ubiquitin ligase complex recognizes its substrates with either D-Box and/or KEN-box motifs (19, 24, 25). Although Aurora-A has four D-Box motifs and one KEN-box motif, the one of four D-box (D-box at C-terminal) and N-terminal A-box (^47^RxLxPSN^52^) are required for the ubiquitylation of human Aurora-A protein (4, 5, 9, 10). Moreover, *Xenopus* Ser53 (or Ser51 in humans) within the A-box is phosphorylated during mitosis, and this phosphorylation is essential for mitotic-specific stabilization (4, 11, 12) (Figure 2). Similarly as Aurora-A regulation via phosphorylation, CDC6 protein is protected from APC/C^Cdh1^-mediated degradation by virtue of its phosphorylation (31). The phosphorylation sites of CDC6 by cyclin E/CDK2 are located directly adjacent to the D-box, therefore preventing the recognition of CDC6 by APC/C^Cdh1^. In the case of human Aurora-A protein, Ser51 is located far from the D-box, but Ser51 is located in the A-box. However, phosphorylated Aurora-A at Ser51 can bind to Cdh1 (4). The mechanism by which Aurora-A degradation is prevented by phosphorylation on Ser51 is unclear. Other regulators, such as Cdc4/Fbxw7, checkpoint with forkhead and ring finger domain (Chfr), and Aurora-A–interacting protein 1 (AIP), are involved in degradation of Aurora-A protein (49–51).

It is well known that overexpression of Aurora-A protein is frequently observed in various human cancers, and that aneuploidy, centrosome amplification, and tumorigenic transformation are induced by its overexpression in cultured human and rodent cells (3, 45, 52). Indeed, *Aurora-A* is mapped to chromosome 20q13.2, a region commonly amplified in human cancers (45, 52, 53). Therefore, Aurora-A overexpression is believed to be caused by gene amplification or transcriptional activation. However, a previous report illustrated that *Aurora-A* amplification was detected in only 3% of cases, but overexpression of Aurora-A mRNA and protein was observed in more than 60% of cases in hepatocellular carcinomas (54). Similar discrepancies between gene amplification and protein overexpression rates of Aurora-A are reported in other types of cancers, including head and neck, breast, gastric, and ovarian (4, 52, 55, 56). Interestingly, constitutive phosphorylation of Ser51 is observed in head and neck cancer cells with overexpression of Aurora-A protein. As Ser51 phosphorylation inhibits APC/C^Cdh1^-mediated degradation, it is possible that constitutive phosphorylation on Ser51 may induce protein stabilization and its consequent accumulation in cancer cells that exhibit overexpression of Aurora-A protein (4). Importantly, Aurora-A overexpression is considered to promote tumorigenesis via disruption of maintenance of the normal centrosome or chromosome number (3, 57, 58).

## DNA Replication and Pre-RC Formation

The ability of a eukaryotic cell to precisely and accurately replicate its DNA is crucial to maintain genome stability. Eukaryotic chromosomes need to be replicated by numerous replication forks that are initiated from replication origins spaced throughout the genome because of the sizes of the chromosomes. Therefore, eukaryotic cells are continually exposed to a risk of over-replication. As described previously, licensing is restricted to occur only once per cell cycle to prevent over-replication. Licensing is the assembly of the pre-RC on replication origins (13, 14). Pre-RC is composed of the origin recognition complex (ORC), cell division cycle 6 (Cdc6), CDT1, and the mini-chromosome maintenance (MCM) proteins (59). Cdc6 and CDT1 are loaded onto replication origins in an ORC-dependent manner during late M and early G_1_ phase, after which they subsequently recruit MCM proteins to the origins. Pre-RC formation occurs from late mitosis to early G_1_. The pre-RC is a protein complex composed of ORC, CDC6, CDT1, and MCM2–7, known as putative DNA helicase (13, 14). During late M and early G_1_, CDC6 and CDT1 bind to replication origins and subsequently induce the recruitment of MCMs to the origins (13, 14). Pre-RC formation is needed for replication in the subsequent S phase. Therefore, it is necessary to prevent re-assembly of the pre-RC during S, G_2_, and M phase. Two major inhibitory pathways exist to prevent pre-RC re-assembly, namely CDK1- and CDK2-based pathways. CDK1 inactivation during G_2_ phase induces re-replication through re-assembly of MCMs (60). Consistently, silencing of cyclin A, but not cyclin B, causes re-replication in *Drosophila* cells (61). Taken together, CDKs suppress re-replication by preventing pre-RC re-assembly. To explain this phenomenon, multiple mechanisms are considered in S and G_2_ phases. For example, CDT1 and ORC1 are phosphorylated by CDKs, resulting in their degradation in an SCF^Skp2^-dependent manner (62–65). Additionally, CDKs phosphorylate CDC6 and induce its nuclear export in mammalian cells (66–68). Another pathway involves geminin, known as an inhibitor of DNA replication. Geminin functionally inhibits pre-RC re-assembly through direct binding to CDT1 during S, G_2_, and M phases, which ensures genome stability and prevents aneuploidy (15). Indeed, ectopic overexpression of geminin suppresses pre-RC formation and subsequently blocks DNA replication (69). In addition, geminin knockdown in mammalian cells induces re-replication (70, 71), indicating that geminin has critical roles in the regulation of replication. Although it seemingly sounds contradictory, geminin stabilizes CDT1 protein during mitosis via preventing its ubiquitin-mediated proteolysis (69). Furthermore, the mitotic depletion of geminin induces CDT1 downregulation and prevents MCM loading in the ensuing G_1_ phase (69, 72). Thereby, the negative and positive roles of geminin are essential for pre-RC formation, indicating that the protein level of geminin must be strictly controlled for proper DNA replication.

## Involvement of Aurora-A in Pre-RC Formation

To ensure pre-RC assembly during late mitosis and early G_1_ phase, cell cycle-dependent degradation of geminin is caused by the UPS (73). The geminin protein level oscillates during the cell cycle via APC/C-mediated ubiquitylation (69, 73) (Figure 1). Recent analyses at the single-cell level by time-lapse fluorescence microscopy analysis revealed that geminin degradation takes place following cyclin B degradation in late anaphase (74). Although Geminin is a substrate of APC/C, geminin is stable even in mitosis in spite of active APC/C. Indeed, geminin is phosphorylated by Aurora-A on Thr25 to prevent its APC/C-dependent proteolysis in mitosis (69) (Figure 1). Geminin contains the consensus sequences (R-X-S/T-L/V) recognized by Aurora-A as observed in amino acids 23–26 (*R*R*TL*) within the D-box of geminin (69). Interestingly, immunoprecipitation analysis revealed that both HA-tagged Cdh1 and HA-tagged Cdc20 interacted with wild-type geminin and Thr25 phospho-defective mutant (geminin^T25A^) but not with Thr25 phospho-mimicking mutant (geminin^T25D^), indicating that the inability of geminin^T25D^ to interact with APC/C^Cdh1^ and APC/C^Cdc20^ may explain its resistance to APC/C-dependent proteolysis (69). In general, distinct substrates are specifically recognized by APC/C complex and are tightly degraded to adjust the critical timing (19). In fact, all of substrates of APC/C are not degraded at same time even though APC/C is active. Phosphorylation in CDC6, Aurora-A, and Skp2 as well as geminin protects from APC/C-mediated ubiquitylation (4, 31, 32). In particular, phosphorylation in CDC6, Skp2, or geminin interferes with the binding of APC/C^Cdh1^ (31, 32, 69). We previously have shown that the phosphorylation of human Aurora-A on Ser51 interferes with its ubiquitylation by APC^Cdh1^. Interestingly, constitutive phosphorylation on Ser51 is well correlated with protein overexpression and stabilization in cancer cells (4). As geminin is frequently overexpressed in certain types of human cancer (75, 76), it is interesting to examine if constitutive phosphorylation at Thr25 induces its protein overexpression in cancer. Importantly, stabilized geminin during mitosis ensures pre-RC formation via protecting CDT1 ubiquitylation by SCF^Skp2^ (69). Aurora-A–geminin–CDT1 axis regulates proper DNA replication (Figure 2).

## Conclusion

Aurora-A is a well-known mitotic kinase that regulates mitotic entry, spindle formation, and chromosome maturation as a canonical role. In this review, we shed light on a novel function of Aurora-A for regulating DNA replication via proper formation of the pre-RC. Indeed, Aurora-A phosphorylates geminin to prevent APC/C-mediated proteolysis in mitosis. To ensure pre-RC formation, stabilized mitotic geminin protects CDT1 from SCF^Skp2^-dependent proteolysis. This novel mechanism controlled by the Aurora-A–geminin–CDT1 axis is essential for proper regulation of DNA replication (Figure 2). Emi1 was identified as a factor inhibiting the function of APC/C^Cdh1^ and it is degraded by SCF^βTrcp^ at early M phase (77–80). It was recently revealed that Emi1 silencing prevents the transition from S to G_2_ phase by downregulating geminin via APC/C activation (81, 82). Therefore, the protein level of geminin is also regulated by the Emi1-mediated inhibition of APC/C^Cdh1^ activity. During cell cycle progression, strict regulation of the amount of geminin protein is essential for proper DNA replication. The protein level of geminin is strictly determined by Emi1- and Aurora-A-mediated protection from ubiquitylation by APC/C.

A series of periodic kinase reactions by CDKs promote cell cycle progression and the fidelity of cell division is dependent on the accumulation and ordered destruction of critical protein regulators (1). Thus, the UPS contributes to the precise regulation of the cell cycle. The UPS also contributes to the precise regulation of DNA replication via the Aurora-A–geminin–CDT1 axis (Figure 2). Interestingly, Aurora-A protein is also ubiquitylated by APC/C^Cdh1^. It is well known that overexpression and/or hyperactivation of Aurora-A is involved in tumorigenesis via aneuploidy and genomic instability (3). Moreover, Aurora-A is frequently overexpressed in various cancers (3, 43, 52–54). As DNA replication is strictly regulated to prevent over-replication in mammalian cells, disruption of this mechanism may be involved in Aurora-A-mediated tumorigenesis. We suggest that deregulation of DNA replication via Aurora-A–geminin–CDT1 axis can be used as a potential diagnostic and therapeutic target in cancer.

## Conflict of Interest Statement

The authors declare that the research was conducted in the absence of any commercial or financial relationships that could be construed as a potential conflict of interest.
